# Successful treatment of extensively drug-resistant *Acinetobacter baumannii* ventriculitis with polymyxin B and tigecycline- a case report

**DOI:** 10.1186/s13756-018-0313-5

**Published:** 2018-02-14

**Authors:** Wei Guo, Shao-Chun Guo, Min Li, Li-Hong Li, Yan Qu

**Affiliations:** Department of Neurosurgery, Tangdu Hospital, Fourth Military Medical University, Xi’an, Shaanxi 710038 China

**Keywords:** *Acinetobacter Baumannii*, Multidrug resistance, Polymyxin B, Ventriculitis, Intraventricular therapy

## Abstract

**Background:**

*Acinetobacter baumannii* nosocomial ventriculitis/meningitis, especially those due to drug-resistant strains, has substantially increased over recent years. However, limited therapeutic options exist for the *Acinetobacter baumannii* ventriculitis/meningitis because of the poor penetration rate of most antibiotics through the blood-brain barrier.

**Case presentation:**

A 57-year-old male patient developed ventriculitis from an extensively drug-resistant strain of *Acinetobacter baumannii* after the decompressive craniectomy for severe traumatic brain injury. The patient was successfully treated with intraventricular and intravenous polymyxin B together with intravenous tigecycline.

**Conclusions:**

The case illustrates intraventricular polymyxin B can be a therapeutic option against extensively drug-resistant *Acinetobacter baumannii* ventriculitis.

## Background

*Acinetobacter baumannii* has emerged as a major nosocomial central nervous system infection, with mortality rates ranging from 15% to 71% for acinetobacter meningitis [1]. Successful treatments with intraventricular/intrathecal polymyxins have been reported; however, the incidence of potential toxicity was not negligible [2]. Here, we report a successful microbiological cure for extensively drug-resistant *A. baumannii* ventriculitis using intravenous and intraventricular polymyxin B together with intravenous tigecycline.

## Case presentation

A 57-year-old male patient was transferred from another hospital to the neurosurgical intensive care unit of the Tangdu hospital. The patient had experienced a falling accident at work and then was diagnosed as having a severe traumatic brain injury. The patient underwent a decompressive craniectomy and an external ventricular drain (EVD) insertion. Eight days after the operation, the patient presented with remittent fever (peak at 39.0 °C) associated with meningeal signs and altered mental status. Empirical antimicrobial therapy was initiated with meropenem and vancomycin. Twelve days after the operation, the patient was transferred to our intensive care unit. A right frontal EVD was inserted because of bilateral hydrocephalus. Cerebrospinal fluid (CSF) analysis revealed a WBC count of 5550 × 10E6/L, with 70% polymorphonuclear leukocytes, a glucose concentration of 1.11 mmol/L, and protein levels of 3662.1 mg/L. CSF sample was cultured on columbia agar with 5% sheep blood at 35 °C in aerobic conditions for 48 h. The bacteria were identified by an automated mass spectrometry microbial identification system (VITEK MS, bioMérieux). On day 5 of the hospitalization, the patient’s CSF culture showed that the *A. baumannii* was susceptible only to polymyxin (MIC = 1 μg/mL) and tigecycline (MIC ≤1 μg/mL). According to the Clinical and Laboratory Standards Institute criteria, polymyxin breakpoints are susceptible (≤2 μg/ml) and resistant (≥4 μg/ml). For tigecycline, the U.S. Food Drug Administration proposed breakpoints are susceptible (≤2 μg/ml), intermediate (4 μg/ml) and resistant (8 μg/ml). A chest computed tomography scan showed lung infiltrates, which were suggestive of pneumonia. Sputum sample was cultured on Columbia agar with 5% sheep blood and MacConkey at 35 °C in aerobic conditions for 48 h. The same strain of *A. baumannii* was isolated from the sputum (polymyxin susceptibility was not tested). The antimicrobial therapy was changed to intraventricular (IVT) polymyxin B (50,000 U q24h), intravenous polymyxin B (450,000 U q12h), and tigecycline (50 mg q12h). The patient became afebrile 5 days after the polymyxin B and tigecycline therapy, with negative CSF cultures thereafter. However, in the treatment process, decreased CSF drainage and a contractible right ventricle were observed gradually. Ten days after the right frontal EVD placement, the right EVD was removed, and another EVD was inserted into the left lateral ventricle. Antimicrobial treatment was switched to IVT polymyxin B (25,000 U q12h), intravenous polymyxin B (475,000 U q12h), and tigecycline (50 mg q12h). A contractible left ventricle was also observed after the IVT polymyxin B administration. Next, the antimicrobial regimen was changed by stopping the IVT polymyxin B administration and continuing the intravenous polymyxin B (500,000 U q12h) and tigecycline (50 mg q12h) administration for another 14 days until the patient’s clinical conditions were stable. On day 31 of the hospitalization, the patient was discharged.

## Discussion and conclusion

Over the years, *Acinetobacter baumannii*, which is associated with post-neurosurgical meningitis and ventriculitis, has increasingly been regarded as an important nosocomially acquired pathogen [1, 3]. Statistical data showed that 3.6–11.2% of post-neurosurgical meningitis cases are caused by *A. baumannii* [2, 4]. Antimicrobial-resistant *A. baumannii* are divided into three categories: multidrug-resistant (MDR), extensively drug-resistant (XDR) and pandrug-resistant (PDR). XDR is defined as non-susceptibility to all penicillins and cephalosporins (including inhibitor combinations), fluroquinolones, aminoglycosides, and carbapenems. [5]. In the present case, XDR *A. baumannii* that was susceptible only to polymyxin and tigecycline was cultured from the cerebral spinal fluid (CSF) and sputum. As a result of the poor blood-brain barrier penetration, intraventricular (IVT) therapy polymyxin B was used through the external ventricular derivation (EVD).

Emerging evidence has indicated that intrathecal (IT) or IVT colistin administration is a safe and effective treatment for XDR *A. baumannii* meningitis [6]. Karaiskos et al. [2]. summarized 36 studies and a total of 81 patients who were diagnosed with meningitis secondary to neurosurgical procedures. A total of 89% (72/81) of the cases treated with IVT/ITH colistin were eventually cured, and the median time to achieve sterilization of the CSF was 4 days. De Bonis et al. [3]. compared the outcomes of the XDR *A. baumannii* ventriculomeningitis patients treated with intravenous (IV) colistin or IV plus IVT colistin. Compared with 33.3% of the cases in the IV alone group, 100% of the cases achieved CSF sterilization (a negative CSF culture result) in the IV + IVT group. The results showed that IVT colistin administration is significantly more effective than IV colistin alone [3]. To the best of our knowledge, polymyxin B and colistin (polymyxin E) were regarded as equivalent because of their similar chemical structures and their activity spectra [7]. However, compared to colistin, there is limited clinical data for the use of polymyxin B in ventriculitis treatment. Piparsania et al [8]. showed successful treatment of multidrug-resistant A. baumannii neonatal meningo-ventriculitis with IVT polymyxin B in combination with IV netilmicin and polymyxin B. IVT polymyxin B (40,000 units per dose) was given alternate day for four weeks. In the present case, sterilization of CSF was detected 5 days after the IVT polymyxin B administration. Hence, we speculate that IVT polymyxin B administration could be as effective as IVT colistin administration.

In the present case, tigecycline was used intravenously. Tigecycline, which belongs to a new class of antibiotics known as the glycylcyclines [9], has demonstrated excellent activity against *Acinetobacter* strains [10]. Pallotto et al. [11] showed the weak penetration of tigecycline to the CSF in a patient with ventriculo-atrial shunt infection. The average CSF concentration of tigecycline equals to 7.9% of the serum concentration. That is the reason why tigecycline is not currently recommended for *Acinetobacter* ventriculitis. Recently, Lauretti et al. reported a successful case of the IVT tigecycline use to treat PDR *A. baumannii* meningitis [12].

Potential toxicity is a concern associated with local administration of polymyxins. Chemical ventriculitis and meningitis which usually cause fever and altered mental state, are the most severe adverse effects reported with IVT/ITH polymyxin treatment. Karaiskos reviewed the literature and found that out of 81 patients with IVT/ITH colistin administration, chemical meningitis and chemical ventriculitis were diagnosed in 3 (3.7%) and 2 (2.4%) cases, respectively [2]. A recent retrospective study showed that out of 9 patients with IVT colistin administration, no cases of chemical meningitis were encountered [3]. However, to the best of our knowledge, limited reports on the adverse effects of IVT/ITH polymyxin B were published.

**Fig. 1:**
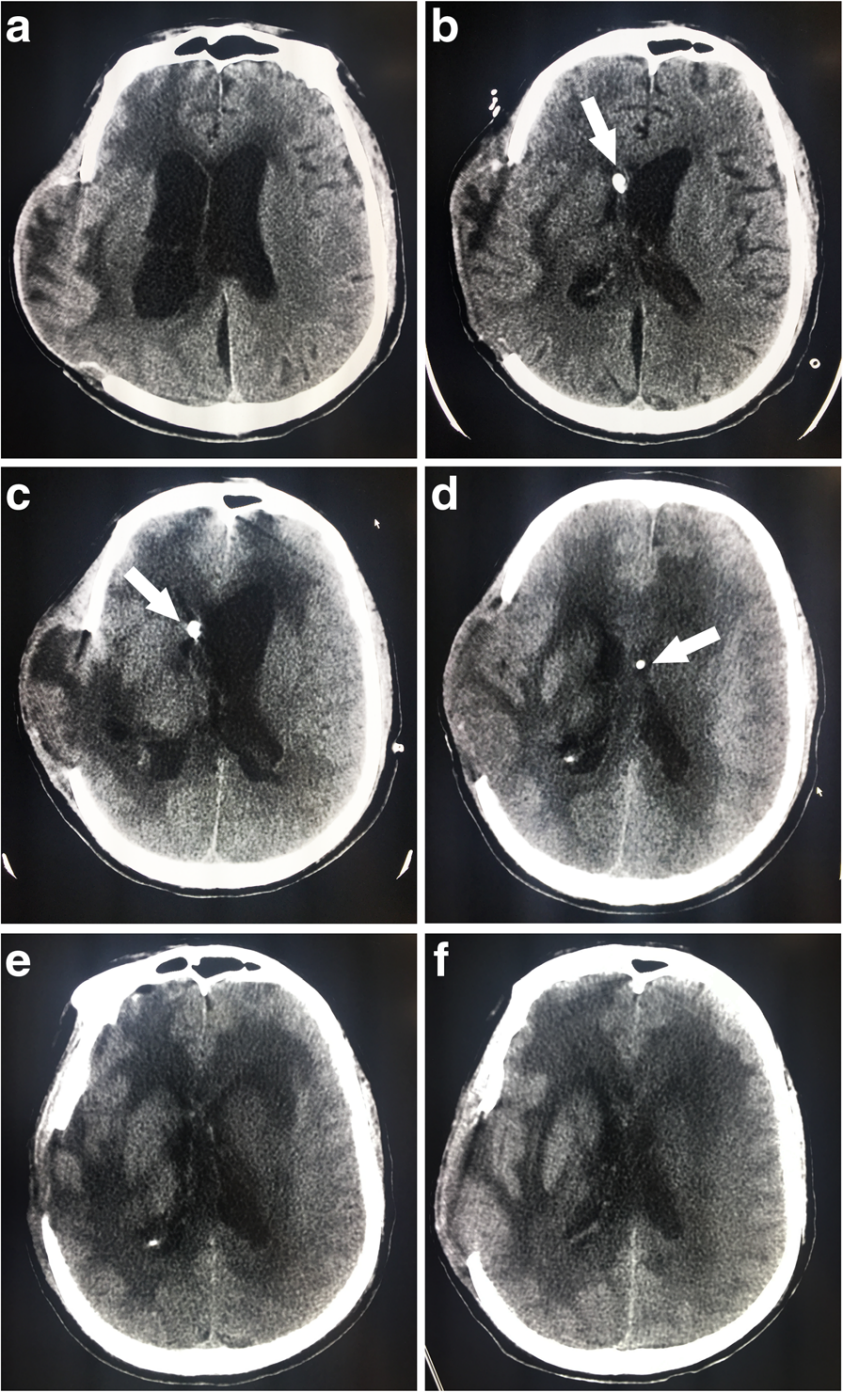
Transient ventricular adhesion due to intraventricular (IVT) polymyxin B administration observed from CT scan. **a** CT scan obtained on day 1 of the hospitalization, showing bilateral hydrocephalus. **b** On day 6 of the hospitalization, CT scan showing the contractible right ventricle and enlarged left ventricle after IVT polymyxin B administration through right EVD (arrows). **c** On day 11 of the hospitalization, CT scan showing the contractible right ventricle and enlarged left ventricle after IVT polymyxin B administration through right EVD (arrows). **d** On day 18 of the hospitalization, CT scan showing the contractible left ventricle after IVT polymyxin B administration through left EVD (arrows). **e** CT scan obtained on day 20 of the hospitalization. **f** CT scan obtained on day 23 of the hospitalization

In the treatment process, two observations were noted. First, the transient ventricular adhesion due to the IVT polymyxin B administration was observed from the CT scan (Fig. 1). Initially, the IVT polymyxin B was administered through the right EVD. However, decreased CSF drainage and a contractible right ventricle were detected after the IVT polymyxin B administration. Six days later, when the left EVD were used for the polymyxin B administration, left ventricle shrinkage was observed. These phenomena indicate that there is a direct correlation between polymyxin B usage and ventricular adhesion. Second, at the 6-month follow-up, the neurological conditions of the patients had not improved, and the patient was still in a comatose status.

Therefore, we conclude that IVT and intravenous polymyxin B combined with intravenous tigecycline could be an effective therapeutic option in the treatment of XDR *A. baumannii* ventriculitis. Currently, IVT administration of antibiotics with favorable outcomes is widely reported. However, multicenter randomized studies are still needed to demonstrate the efficacy and safety of intraventricular administration on these patients.

## Abbreviations

CSF: Cerebrospinal fluid
EVD: External ventricular drain
IT: Intrathecal
IV: Intravenous
IVT: Intraventricular
MDR: Multidrug-resistant
PDR: Pandrug-resistant
XDR: Extensively drug-resistant

## References

[1] Kim BN, Peleg AY, Lodise TP, Lipman J, Li J, Nation R, Paterson DL (2009). Management of meningitis due to antibiotic-resistant Acinetobacter species. Lancet Infect Dis.

[2] Karaiskos I, Galani L, Baziaka F, Giamarellou H. Intraventricular and intrathecal colistin as the last therapeutic resort for the treatment of multidrug-resistant and extensively drug-resistant Acinetobacter baumannii ventriculitis and meningitis: a literature review. Int J Antimicrob Agents. 41(6):499–508. 10.1016/j.ijantimicag.2013.02.006 23507414

[3] De Bonis P, Lofrese G, Scoppettuolo G, Spanu T, Cultrera R, Labonia M, Cavallo MA, Mangiola A, Anile C, Pompucci A. Intraventricular versus intravenous colistin for the treatment of extensively drug resistant Acinetobacter Baumannii meningitis. Eur J Neurol. 23(1):68–75. 10.1111/ene.12789 26228051

[4] Wang KW, Chang WN, Huang CR, Tsai NW, Tsui HW, Wang HC, Su TM, Rau CS, Cheng BC, Chang CS (2005). Post-neurosurgical nosocomial bacterial meningitis in adults: microbiology, clinical features, and outcomes. J Clin Neurosci.

[5] Magiorakos AP, Srinivasan A, Carey RB, Carmeli Y, Falagas ME, Giske CG, Harbarth S, Hindler JF, Kahlmeter G, Olsson-Liljequist B. Multidrug-resistant, extensively drug-resistant and pandrug-resistant bacteria: an international expert proposal for interim standard definitions for acquired resistance. Clin Microbiol Infect. 18(3):268–81. 10.1111/j.1469-0691.2011.03570.x 21793988

[6] Hoenigl M, Drescher M, Feierl G, Valentin T, Zarfel G, Seeber K, Krause R, Grisold A. Successful management of nosocomial ventriculitis and meningitis caused by extensively drug-resistant Acinetobacter baumannii in Austria. Can J Infect Dis Med Microbiol. 24(3):e88–90. 10.1155/2013/613865 PMC3852464 24421838

[7] Cai Y, Lee W, Kwa AL. Polymyxin B versus colistin: an update. Expert Rev Anti-Infect Ther. 13(12):1481–97. 10.1586/14787210.2015.1093933 26488563

[8] Piparsania S, Rajput N, Bhatambare G (2012). Intraventricular polymyxin B for the treatment of neonatal meningo-ventriculitis caused by multi-resistant Acinetobacter Baumannii– case report and review of literature. Turk J Pediatr.

[9] Pankey GA (2005). Tigecycline. J Antimicrob Chemother.

[10] Karageorgopoulos DE, Falagas ME (2008). Current control and treatment of multidrug-resistant Acinetobacter Baumannii infections. Lancet Infect Dis.

[11] Pallotto C, Fiorio M, D'Avolio A, Sgrelli A, Baldelli F, Di Perri G, De Socio GV. Cerebrospinal fluid penetration of tigecycline. Scand J Infect Dis. 46(1):69–72. 10.3109/00365548.2013.837957 24131423

[12] Lauretti L, D'Alessandris QG, Fantoni M, D'Inzeo T, Fernandez E, Pallini R, Scoppettuolo G (2016). First reported case of intraventricular tigecycline for meningitis from extremely drug-resistant Acinetobacter Baumannii. J Neurosurg.

